# Results from Arm A of Phase 1/2 DREAMM-6 trial: belantamab mafodotin with lenalidomide plus dexamethasone in patients with relapsed/refractory multiple myeloma

**DOI:** 10.1038/s41408-024-01155-y

**Published:** 2024-10-21

**Authors:** Rakesh Popat, Bradley Augustson, Mercedes Gironella, Cindy Lee, Paul Cannell, Nashita Patel, Ravi S. Kasinathan, Rachel Rogers, Mehreen Shaikh, Amy Curry, Fernando Carreño, Sumita Roy-Ghanta, Joanna Opalinska, Hang Quach

**Affiliations:** 1 NIHR UCLH Clinical Research Facility, University College London Hospitals NHS Foundation Trust, London, UK; 2 Sir Charles Gairdner Hospital and Linear Clinical Research, Perth, WA Australia; 3 Department of Hematology, University Hospital Vall d’Hebron, Barcelona, Spain; 4 Royal Adelaide Hospital, Adelaide, SA Australia; 5 Department of Haematology, Fiona Stanley Hospital, Perth, WA Australia; 6 GSK, London, UK; 7 GSK, Upper Providence, PA USA; 8 GSK, Durham, NC USA; 9 University of Melbourne, St. Vincent’s Hospital Melbourne, Melbourne, VIC Australia

**Keywords:** Diseases, Targeted therapies

## Abstract

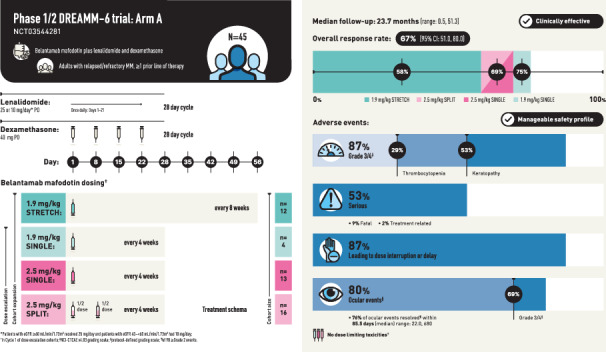

Belantamab mafodotin is a first-in-class humanized, afucosylated, anti-B-cell maturation antigen (BCMA) monoclonal antibody conjugated to the microtubule inhibitor monomethyl auristatin F [1]. Preclinical studies showed belantamab mafodotin interacts synergistically with standard-of-care agents for multiple myeloma (MM) including bortezomib, lenalidomide, pomalidomide, and dexamethasone to enhance anti-tumor activity [1, 2]. A number of clinical studies are assessing belantamab mafodotin combination therapies in patients with MM [3–10]. The Phase 3 DREAMM-7 (NCT04246047) trial showed that patients with relapsed/refractory MM (RRMM) who received ≥1 prior line of therapy (LOT) had a significant improvement of progression-free survival (PFS) with belantamab mafodotin plus bortezomib and dexamethasone versus daratumumab plus bortezomib and dexamethasone [10]. Similarly, a prespecified interim analysis of the Phase 3 DREAMM-8 (NCT04484623) trial showed a significantly extended PFS. A positive trend in overall survival (OS) was also demonstrated in both studies [9].

We report results from Arm A of the Phase 1/2 DREAMM-6 (NCT03544281) trial of belantamab mafodotin in combination with lenalidomide plus dexamethasone in patients with RRMM who received ≥1 prior LOT (Table S1). The design of Arm A is detailed in Fig. S1 and consists of dose escalation and dose expansion parts. For dose escalation, the primary endpoints were the number and proportion of patients with dose-limiting toxicities (DLT) during Cycle 1 and adverse events (AE), including serious AE (SAE). For dose expansion, AE, SAE, and overall response rate (ORR) were primary endpoints. ORR was defined as the proportion of patients achieving a partial response (PR) or better, according to IMWG response criteria [11], and was assessed every 4 weeks (±3 days). Four belantamab mafodotin dosing cohorts were assessed: belantamab mafodotin administered at 1.9 mg/kg and 2.5 mg/kg as a single dose on Day 1 every 4 weeks (Q4W, SINGLE cohorts), 2.5 mg/kg administered as a 50:50 split dose on Day 1 and Day 8 (SPLIT cohort), or 1.9 mg/kg every 8 weeks as a single dose on Day 1 (STRETCH cohort). Full lenalidomide and dexamethasone dosing schedules are provided in the Supplementary Methods.

AE of special interest (AESI) were secondary safety endpoints, and included ocular events, thrombocytopenia, infusion-related reactions, and ocular findings on ophthalmic exam. Ocular event assessments included event frequency, severity, time-to-first event, event type, and resolution of event (by final analysis and at last visit) and were graded either using the NCI-CTCAE v4.03 or a protocol-defined scale that captures both corneal findings and visual acuity changes based on best corrected visual acuity (BCVA) score and slit lamp findings (per the criteria detailed in Table S2).

Exploratory efficacy outcomes (dose-escalation and -expansion parts) included: complete response (CR) rate (CRR), rate of minimal residual disease (MRD) negativity, duration of response, and PFS.

Forty-five patients were included in the all-treated analysis population, with 12 patients in the 1.9 mg/kg STRETCH, 4 in the 1.9 mg/kg SINGLE, 13 in the 2.5 mg/kg SPLIT, and 16 in the 2.5 mg/kg SINGLE cohorts (Fig. S2). The all-treated population had a median (range) age of 68 (36–80) years, with 18% of patients aged ≥75 years. Most patients were male (78%). Overall, 80% of patients had an International Staging System stage of I or II; patients received a median (range) number of 3.0 (1–10) prior LOT, with 58% and 31% having prior lenalidomide and daratumumab exposure, respectively (Table S3). Median (range) follow-up was 23.7 (0.5, 51.3) months in the all-treated population and 22.9 (0.5, 26.8) months in the 1.9 mg/kg STRETCH, 27.7 (15.2, 41.3) months in the 1.9 mg/kg SINGLE, 30.6 (2.0, 35.6) months in the 2.5 mg/kg SPLIT, and 27.9 (0.5, 51.3) months in the 2.5 mg/kg SINGLE cohorts.

In the dose-escalation part, no DLTs were reported in any cohort. In the all-treated population, there were no clinically meaningful differences in the overall safety profile across the cohorts (Table 1). AEs were reported in all patients, with the most common Grade 3/4 being keratopathy (53%), decreased neutrophil count (22%), decreased platelet count (22%), and reduced visual acuity (22%; Table 1). Infections occurred in 67% of patients, and most were Grade 1–2; the only Grade ≥3 infection that occurred in 2 or more patients in any arm was pneumonia (16%).

**Table 1:** Safety summary.

	1.9 mg/kg STRETCH (*n* = 12)	1.9 mg/kg SINGLE (*n* = 4)	2.5 mg/kg SPLIT (*n* = 13)	2.5 mg/kg SINGLE (*n* = 16)	All-treated (*N* = 45)
**Any AE,** ***n*** **(%)**	12 (100)	4 (100)	13 (100)	16 (100)	45 (100)
Related to any study treatment	11 (92)	4 (100)	13 (100)	15 (94)	43 (96)
Leading to any study treatment discontinuation	3 (25)	1 (25)	5 (38)	3 (19)	12 (27)
Leading to dose interruption/delay	11 (92)	3 (75)	12 (92)	13 (81)	39 (87)
Grade 3 or 4	10 (83)	3 (75)	13 (100)	13 (81)	39 (87)
Related to any study treatment	7 (58)	3 (75)	12 (92)	13 (81)	35 (78)
≥20% incidence					
Keratopathy^a^	6 (50)	2 (50)	7 (54)	9 (56)	24 (53)
Neutrophil count decreased	3 (25)	1 (25)	2 (15)	4 (25)	10 (22)
Platelet count decreased	2 (17)	0	5 (38)	3 (19)	10 (22)
Visual acuity reduced	1 (8)	1 (25)	3 (23)	5 (31)	10 (22)
**Non-ocular AESI,** ***n*** **(%)**					
Any thrombocytopenia event^b^	4 (33)	2 (50)	7 (54)	11 (69)	24 (53)
Grade 3 or 4	2 (17)	1 (25)	5 (38)	5 (31)	13 (29)
Any infusion-related reactions	1 (8)	0	2 (15)	3 (19)	6 (13)
Grade 3 or 4	0	0	0	0	0
**Ocular AE**					
Protocol-defined grading, any event	9 (75)	3 (75)	12 (92)	12 (75)	36 (80)
Grade 3 or 4	8 (67)	3 (75)	10 (77)	10 (63)	31 (69)
Time to first Grade ≥2 event, days, median (range)	29.0 (25.0, 57.0)	29.0 (29.0, 42.0)	41.5 (23.0, 59.0)	30.0 (24.0, 60.0)	n/c
NCI-CTCAE v4.03 graded, any event	9 (75)	3 (75)	12 (92)	11 (69)	35 (78)
Grade 3 or 4	6 (50)	2 (50)	9 (69)	9 (56)	26 (58)
Related to belantamab mafodotin, *n* (%)	9 (75)	3 (75)	12 (92)	11 (69)	35 (78)
NCI-CTCAE v4.03 defined event type^c^ *n* (%)					
Keratopathy	9 (75)	3 (75)	12 (92)	11 (69)	35 (78)
Visual acuity reduced	5 (42)	1 (25)	4 (31)	5 (31)	15 (33)
Vision blurred	0	1 (25)	7 (54)	6 (38)	14 (31)
Foreign body sensation in eyes	2 (17)	0	2 (15)	1 (6)	5 (11)
Eye pain	1 (8)	0	1 (8)	2 (13)	4 (9)
Dry eye	1 (8)	1 (25)	0	1 (6)	3 (7)
Visual acuity tests abnormal	0	0	2 (15)	0	2 (4)
Protocol-defined Grade ≥2 events^d^	*n* = 30	*n* = 5	*n* = 28	*n* = 35	*n* = 98
Resolved,^e^ *n* (%)	24 (80)	3 (60)	20 (71)	27 (77)	74 (76)
Duration of event, days, median (range)	57.5 (27.0, 476)	281.0 (57.0, 533)	117.5 (29.0, 498)	92.0 (22.0, 680)	85.5 (22.0, 680)
**Any SAE,** ***n*** **(%)**	6 (50)	2 (50)	6 (46)	10 (63)	24 (53)
Related to any study treatment	2 (17)	1 (25)	3 (23)	4 (25)	10 (22)
Fatal	0	0	1 (8)	3 (19)	4 (9)
Related to belantamab mafodotin and lenalidomide	0	0	0	1 (6)	1 (2)

The protocol-defined scale for ocular AE captures both ocular findings and visual acuity changes based on the best corrected visual acuity score and slit lamp findings.

^a^MedDRA Preferred Term.

^b^including reduced platelet count.

^c^reported in >10% of patients with ocular events in at least one cohort.

^d^where data on event resolution status were provided.

^e^by final analysis.

*AE* adverse event, *AESI* adverse event of special interest, *MedDRA* Medical Dictionary for Regulatory Activities, *n/c* not calculated, *NCI-CTCAE* National Cancer Institute-Common Toxicity Criteria for Adverse Events, *SAE* serious adverse event.

The most frequent non-ocular AESI experienced by patients were thrombocytopenias (53%), with Grade ≥3 events occurring in 29% of patients (Table 1); none of these were deemed serious or led to the withdrawal of study treatment. Dose modifications due to thrombocytopenia were not required in 47% of patients, and most patients (58%) had fully or partially recovered from the event by final analysis.

Ocular AEs, per protocol-defined grading scale, were reported in 80% of patients, with 69% of patients having Grade ≥3 events (Table 1). The most common NCI-CTCAE v4.03 defined ocular events were keratopathy (78%), reduced visual acuity (33%), and blurred vision (31%) (Table 1). At the time of final analysis, 35 (78%) patients had experienced a total of 98 protocol-defined Grade ≥2 ocular events. Median time to first protocol-defined Grade ≥2 ocular events ranged from 29.0–41.5 days across the cohorts, with the longest time being in the 2.5 mg/kg SPLIT group (Table 1). A post hoc analysis showed 76% of events (74/98) resolved by final analysis, over a median (range) duration of 85.5 (22.0, 680) days overall, with the shortest median time to resolution in the 1.9 mg/kg STRETCH cohort at 57.5 (27.0, 476) days (Table 1). Overall, 23 patients (51% of all-treated) had ongoing protocol-defined Grade ≥2 ocular events as of the last follow-up.

SAEs were reported in 53% of patients (Table 1). SAEs were fatal in four patients, with one (febrile neutropenia) deemed related to belantamab mafodotin and lenalidomide treatment in the 2.5 mg/kg SINGLE cohort; SAEs of pneumonia and sepsis, not related to study treatment, were also reported for this patient. Other fatal SAEs not related to the study treatment included pneumonia due to COVID-19 in one patient in the 2.5 mg/kg SINGLE cohort, and pneumonia in two patients: one each in the 2.5 mg/kg SINGLE and 2.5 mg/kg SPLIT cohorts.

ORR (post hoc) was 67% (95% CI: 51.0, 80.0) and was generally similar across the cohorts (Fig. 1). CRR (post hoc) was 29% (95% CI: 16.4, 44.3) (Fig. 1). MRD negativity rates for patients with a ≥CR and those with a ≥very good partial response were 15.6% (*n* = 7/45) and 22.2% (*n* = 10/45), respectively (results by cohort shown in Table S4). Median PFS was 18.4 months (95% CI: 6.8, NR) for the all-treated population (post hoc) and was not reached in the 1.9 mg/kg STRETCH and 2.5 mg/kg SINGLE cohorts. Additional data (pharmacokinetics, exposure-response, response by soluble BCMA level, anti-drug antibodies, health-related quality of life), are reported in the Supplemental Results.

**Fig. 1:**
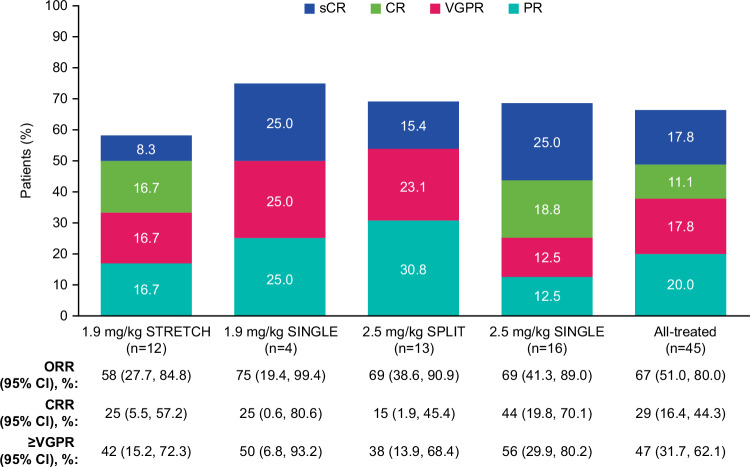
ORR. ORR was defined as a PR or better and CRR was defined as a confirmed CR or better; patients with unknown or missing responses were treated as non-responders. Deep responses are indicated as ≥VGPR. CI confidence interval; CR complete response; CRR complete response rate; ORR overall response rate; PR partial response; s stringent; VGPR very good partial response. From Belantamab Mafodotin in Combination with Lenalidomide Plus Dexamethasone in Patients with Relapsed/Refractory Multiple Myeloma: Safety and Efficacy Analysis of DREAMM-6 Trial Arm-A/#2010, presented by Rakesh Popat at the 2023 ASH Annual Meeting. Reproduced with permission.

Results from Arm A of the DREAMM-6 study showed no DLT or new safety signals across cohorts. AEs, including ocular events, were generally consistent with the safety profiles of the individual agents, with no clinically meaningful differences across the belantamab mafodotin dosing cohorts. However, comparisons are limited by the small sample size. The overall safety profile of the belantamab mafodotin treatment combination was generally consistent with the Phase 2 DREAMM-2 (NCT03525678) and the Phase 3 DREAMM-3 (NCT04162210) studies evaluating belantamab mafodotin monotherapy in heavily pretreated patients with RRMM [12, 13]. Keratopathy was the most frequent NCI-CTCAE Grade 3/4 AE reported for DREAMM-6 Arm A, occurring in approximately half of patients, a rate consistent with those reported by other clinical studies assessing belantamab mafodotin Q4W in combination regimens [4, 6]. Treatment with belantamab mafodotin across the range of doses/schedules in combination with lenalidomide and dexamethasone had a manageable safety profile for patients with RRMM.

ORR was broadly similar across cohorts, taking into consideration the different lengths of median follow-up (22.9–30.6 months), and was consistent with other belantamab mafodotin combination studies [4, 6, 8, 10]. Of note, responses were observed in this population with 58% prior lenalidomide exposure. Efficacy by lenalidomide-refractory status is not available for this study, as data on refractory status of prior lines was not collected. These data would be of interest for future studies, as first-line lenalidomide use has increased the number of patients who are refractory in early therapy lines. Further, belantamab mafodotin with lenalidomide and dexamethasone is also being investigated in newly diagnosed transplant-ineligible patients, with encouraging preliminary results [14].

In a RRMM population with previous lenalidomide exposure, all tested doses and schedules of belantamab mafodotin with lenalidomide and dexamethasone resulted in clinically meaningful anti-myeloma activity. Further, the safety profile was consistent with belantamab mafodotin monotherapy (including manageable, reversible ocular events). Although our data were not powered to recommend a particular dose or schedule and are limited by the small sample size, ocular events in the 1.9 mg/kg STRETCH regimen had a more rapid resolution while maintaining comparable efficacy to the other regimens. Additionally, patients in the 1.9 mg/kg STRETCH group achieved the highest relative dose intensity for belantamab mafodotin post-dose 1 (80%) compared with the other cohorts (51–68%). These results indicate that dose modifications are effective in managing belantamab mafodotin-related safety, without evident loss in efficacy, but further studies are required.

## Supplementary information

Supplemental Material

## Data Availability

GSK makes available anonymized individual participant data and associated documents from interventional clinical studies that evaluate medicines, upon approval of proposals submitted to: https://www.gsk-studyregister.com/en.
