# Supplementary material for: Results from Arm A of Phase 1/2 DREAMM-6 trial: belantamab mafodotin with lenalidomide plus dexamethasone in patients with relapsed/refractory multiple myeloma
Source: Blood Cancer J. 2024 Oct 21;14(1):184. doi: 10.1038/s41408-024-01155-y (PMC11494064; doi:10.1038/s41408-024-01155-y)
Supplement: Supplementary file 1 — Supplemental Material [file 41408_2024_1155_MOESM1_ESM.docx]

# Results from Arm A of Phase 1/2 DREAMM-6 Trial: Belantamab Mafodotin With Lenalidomide Plus Dexamethasone in Patients With Relapsed/Refractory Multiple Myeloma

Rakesh Popat, Bradley Augustson, Mercedes Gironella, Cindy Lee, Paul Cannell, Nashita Patel, Ravi S. Kasinathan, Rachel Rogers, Mehreen Shaikh, Amy Curry, Fernando Carreño, Sumita Roy-Ghanta, Joanna Opalinska, Hang Quach

# Supplementary materials

### **Supplemental Methods**

#### Institutional and/or licensing committee approval

**Australia**
Bellberry Human Research Ethics Committees; South Metropolitan Health Service Human Research Ethics Committee (RGO); Peter MacCallum Cancer Centre Ethics Committee (RGO); Central Adelaide Local Health Network Incorporated operating as The Queen Elizabeth Hospital (RGO); University of Wollongong and South Eastern Sydney and Illawarra Area Health Service Health; St Vincent's Hospital, HREC D (RGO); The Alfred Hospital Research Governance (RGO).

**Canada**McGill University Health Center-Research Ethics Board.

**France**Comité de Protection des Personnes Ile de France II.

**Spain**CEIC Hospital Universitario Madrid Monteprincipe.

**United Kingdom**South West - Central Bristol Research Ethics Committee.

**United States**Western Institutional Review Board; Michigan State University Biomedical and Health Institutional Review Board; Greenville Health System Institutional Review Board; Memorial Sloan-Kettering Cancer Center Institutional Review Board; Biomedical Research Alliance of New York, LLC (BRANY); Copernicus Group Institutional Review Board; Washington University in Saint Louis Institutional Review Board; Mary Crowley Medical Research Center Institutional Review Board.

#### Lenalidomide and dexamethasone dosing schedule

Lenalidomide was administered orally on Days 1–21 of each 28-day cycle, with doses varying based on patient’s estimated glomerular filtration rate (eGFR); those with eGFR ≥60 mL/min/1.73m^2^ received 25 mg/day, and those with eGFR 40–<60 mL/min/1.73m^2^ had a reduced dose of 10 mg/day. Dexamethasone was administered orally (40 mg) on Days 1, 8, 15, and 22 of each 28-day cycle; at the investigator’s discretion, patients aged >75 years with a body mass index <18.5 kg/m^2^ were eligible to receive a reduced oral dexamethasone dose (20 mg). In the case of intolerable adverse events related to combined lenalidomide and dexamethasone, patients were allowed to continue on belantamab mafodotin monotherapy.

#### Belantamab mafodotin modified Toxicity Probability Interval design and dose-limiting toxicity criteria

Cohorts were recruited in blocks of three patients, with a staggered treatment approach taken (­≥1 day between each patient’s first dose) to minimize the risk of inadvertently exceeding the maximum tolerated dose (MTD) in multiple patients. The design assumed the true underlying toxicity rate for belantamab mafodotin MTD fell within the range from 25% to 35% and centered at 30%. DLT were evaluated in patients receiving ≥1 full dose of belantamab mafodotin and ≥75% of the planned lenalidomide and dexamethasone doses or in patients with DLT in Cycle 1 before receiving a full dose of belantamab mafodotin or <75% of the of the planned lenalidomide and dexamethasone doses. Data from at least 3 (DLT)-evaluable patients were required before a decision was made to enroll patients at the same dose level in the dose escalation part of the study. DLT were assessed using National Cancer Institute Common Terminology Criteria for Adverse Events (NCI-CTCAE) v 4.03 and included at least one of the following: Grade ≥3 febrile neutropenia lasting >48 h despite adequate treatment; Grade 4 thrombocytopenia <25 000/mm^3^ accompanied by clinically significant bleeding; any Grade ≥3 non-hematologic toxicity (other than ocular events) that was more severe than expected with belantamab mafodotin treatment or did not resolve with appropriate supportive treatment within 48 h; any Grade ≥3 non-hematologic laboratory value if the laboratory abnormality persisted for >48 h despite supportive treatment or the abnormality led to hospitalization; any Grade 4 ocular event; or liver toxicity meeting a predefined stopping criteria.

#### Protocol amendments

Overall, four amendments to the study protocol were made. Key changes made in the first amendment included a minor correction to one of the patient eligibility criterion (exclusion of patients with prior allogeneic stem cell transplant to avoid unpredictable toxicity) and clarification of scenarios for study drug dose delays after Cycle 1 to provide guidance on dose interruptions and reductions. Due to a pause in study enrollment in December 2018 (per protocol), owing to the death of the first two patients enrolled (due to neutropenic/infection events), key changes in the second protocol amendment included a change in Eastern Cooperative Oncology Group eligibility criteria from 0–2 to 0­–1; the inclusion of additional guidance for the management of neutropenia/prophylaxis of infections; more stringent hematological monitoring; and the modification of the study design to generate additional safety, pharmacokinetics (PK), and clinical activity data at lower belantamab mafodotin dose levels and the introduction of the 2.5 mg/kg SPLIT cohort (full dose divided into two administrations: 50% of the dose each on Day 1 and Day 8 of a 4-week cycle). For the third protocol amendment, following a review of the safety data from DREAMM-2, which indicated ocular changes associated with belantamab mafodotin treatment were mostly limited to the corneal epithelium (1-3), subsequent collection of ocular safety data was focused on ocular changes, and the use of corticosteroid eye drops as prophylactic treatment was discontinued due to evidence that this treatment is ineffective in preventing or mitigating changes to the corneal epithelium (4). In addition, to reduce increased exposure of belantamab mafodotin over time and to potentially improve the benefit/risk for participants, an extended dosing schedule for belantamab mafodotin was introduced (1.9 mg/kg STRETCH cohort: full dose on Day 1, over an 8-week period). The fourth amendment to the protocol was made to update the definition of the end of study and to include continued details on study intervention access after the final analysis.

### **Supplemental Results**

### PK, exposure-response, sBCMA, and anti-drug antibodies

Population PK analysis showed that the belantamab mafodotin monotherapy PK model adequately described the ADC PK profiles of patients receiving belantamab mafodotin in combination with lenalidomide and dexamethasone. In PK analyses of Cycle 1 on Day 1, belantamab mafodotin maximum concentration (C_max_) values were lower for the 2.5 mg/kg SPLIT cohort than the other cohorts. Average belantamab mafodotin dose concentrations over the 28 days in Cycle 1 (C_avg_28 days_) largely overlapped among cohorts, with a trend suggesting a modest increase in exposure for the 2.5 mg/kg cohorts, within the variability of the data.

In the three cohorts where PK analysis was performed for lenalidomide 25 mg, geometric mean C_max_ (% CV) on Day 1 of Cycle 1 was numerically highest in the 1.9 mg/kg STRETCH group (382.2 [54.5] ng/ml; n=5), followed by the 1.9 mg/kg SINGLE group (331.5 [17.9] ng/ml; n=4), with the lowest C_max_ in the 2.5mg/kg SINGLE cohort (283.1 [36.6] ng/ml; n=13). PK results for the lenalidomide 10 mg dose (n=6) were as anticipated (data not shown).

For the exposure-efficacy analysis, a positive trend was observed between belantamab mafodotin C_avg_28 days_ exposure and the probability of achieving an overall response; the dosing schedule was not found to be a significant covariate. A similar positive trend was seen for the exposure-safety analysis between the probability of NCI-CTCAE–defined Grade ≥2 ocular events and belantamab mafodotin exposure measures. However, for both analyses these associations did not reach statistical significance.

Within the context of the small sample size, baseline median sBCMA levels overlapped between responders and non-responders for all cohorts except 2.5 mg/kg SINGLE; responders versus non-responders had higher median (range) baseline sBCMA levels in the 1.9 mg/kg SINGLE (33.4 [15, 92] vs 18.8 [19, 19]) and 2.5 mg/kg SPLIT (39.6 [8, 106] vs 31.8 [9, 60]) cohorts and lower baseline sBCMA levels in the 1.9 mg/kg STRETCH (23.8 [7, 92] vs 77.5 [7, 308]) and 2.5 mg/kg SINGLE (21.2 [2, 238] vs 515.2 [303, 1185]) cohorts. No patients developed post-treatment anti-drug antibodies to belantamab mafodotin.

### HRQoL

In patients who responded to the PRO-CTCAE questionnaire, maximum post-baseline PRO-CTCAE scores of 3 or 4 for pain were reported in 55.5% of patients for frequency (frequently/almost constantly; range across cohorts: 62–75%), and 37.8% for both severity (severe/very severe; range across cohorts: 33–71%) and interference (quite a bit/very much; range across cohorts: 42–57%); no consistent trends in scores were observed across the cohorts.

Maximum post-baseline PRO-CTCAE scores of 3 or 4 for fatigue were reported in 31.1% of patients for severity (range across cohorts: 15–63%), and 33.3% for interference (range across cohorts: 25–63%); frequency was not applicable. The 1.9 mg/kg STRETCH cohort included the greatest proportion of patients reporting scores of 3 or 4 for fatigue severity (63%, n=5/8) and interference (63%, n=5/8) scores, followed by the 2.5 mg/kg SINGLE cohort (46%, n=6/13 and 46%, n=6/13, respectively).

For HRQoL outcomes related to ocular AE, baseline median OSDI vision-related function score was 0.0–4.2 across cohorts. For worst-case post-baseline score, 25 patients (56%) experienced a minimally clinically meaningful deterioration (≥12.5-point increase in OSDI score) in vision-related function (range across cohorts: 67–90%), with the median worst case post-baseline change in OSDI total scores across cohorts of 19.0–56.8. The 2.5 mg/kg SPLIT cohort had the numerically greatest median (range) worst case post-baseline increase in total score (56.8 [0, 82]), with the 1.9 mg/kg SINGLE having the smallest increase in score (19.0 [0, 33]). However, after the first clinically meaningful worsening in vision-related function, 20 of these patients (80%) reported a clinically meaningful improvement (≥12.5-point decrease in OSDI score) in their vision-related functioning by final analysis: 3 of 4 patients (75%) in the 1.9 mg/kg STRETCH, 3 of 3 (100%) in the 1.9 mg/kg SINGLE, 8 of 9 (89%) in the 2.5 mg/kg SPLIT, and 6 of 9 (67%) in the 2.5 mg/kg SINGLE cohorts.

At baseline, the median NEI-VFQ-25 overall composite score was 87.0–99.2 across cohorts. The median worst case post-baseline actual score was 55.3–79.8 across cohorts, equating to a change in overall composite score over time of -34.4 to -12.7 (i.e. a deterioration); the smallest deterioration in vision-related functioning was seen in the 1.9 mg/kg STRETCH cohort (-12.7 [range: -50, -3]).

### **References**

1. Nooka A, Cohen A, Lee H, Badros A, Suvannasankha A, Callander N, et al. P31SINGLE-AGENT BELANTAMAB MAFODOTIN IN PATIENTS WITH RELAPSED OR REFRACTORY MULTIPLE MYELOMA: FINAL ANALYSIS OF THE DREAMM-2 TRIAL. Hemasphere. 2023;7(Suppl):27-8.

2. Farooq AV, Degli Esposti S, Popat R, Thulasi P, Lonial S, Nooka AK, et al. Corneal Epithelial Findings in Patients with Multiple Myeloma Treated with Antibody-Drug Conjugate Belantamab Mafodotin in the Pivotal, Randomized, DREAMM-2 Study. Ophthalmology and therapy. 2020;9(4):889-911.

3. Nooka AK, Cohen AD, Lee HC, Badros A, Suvannasankha A, Callander N, et al. Single-agent belantamab mafodotin in patients with relapsed/refractory multiple myeloma: Final analysis of the DREAMM-2 trial. Cancer. 2023;129(23):3746-60.

4. Lonial S, Lee HC, Badros A, Trudel S, Nooka AK, Chari A, et al. Belantamab mafodotin for relapsed or refractory multiple myeloma (DREAMM-2): a two-arm, randomised, open-label, phase 2 study. Lancet Oncol. 2020;21(2):207-21.

### **Table S1. Full patient eligibility criteria**

| **Inclusion criteria; at screening, patients:** |
| --- |
| Were capable of giving signed informed consent and complied with the requirements and restrictions listed in the informed consent form |
| Were 18 years or older at the time consent was obtained |
| Had a confirmed MM diagnosis as defined by the International Myeloma Working Group criteria |
| Had an Eastern Cooperative Oncology Group performance status between 0 and 1 |
| Had prior autologous SCT >100 days before study enrollment, with no active bacterial, viral, or fungal infection(s), or were considered transplant ineligible |
| Were treated with ≥ 1 prior LOT and had documented disease progression during or after their most recent therapy |
| Had measurable MM disease defined by ≥1 of the following criteria:   - Urine M-protein excretion ≥200 mg/24 h - Serum M-protein concentration ≥0.5 g/dL - Serum FLC assay FLC level ≥10 mg/dL and an abnormal serum FLC ratio (<0.26 or >1.65) |
| Prior treatment-related toxicities as defined by NCI-CTCAE, Version 4.03, 2010 must have been Grade ≤1 at the time of enrollment, except for alopecia and for patients with Grade 2  neuropathy |
| Had adequate organ system functions as defined by absolute neutrophil count* ≥1.5 × 10^9^/L, hemoglobin ≥8.0 g/dL, platelets ≥75 × 10^9^/L, total bilirubin ≤1.5 × ULN,^†^ ALT ≤2.5 × ULN, eGFR^‡^ ≥40 mL/min/1.73 m^2^, albumin/creatinine ratio from spot urine ≤500 mg/g, left ventricular ejection fraction by echocardiogram ≥40% |
| Were either not a woman of childbearing potential or were using a highly effective (failure rate <1%/year) contraceptive method |
| **Patients not meeting the inclusion criteria were excluded, along with those meeting the following exclusion criteria:** |
| Had prior treatment with a monoclonal antibody within 30 days of receiving the first belantamab mafodotin dose |
| Had prior allogenic SCT; patients with a prior syngeneic transplant were allowed provided there was no history of or active graft versus host disease |
| Had evidence of active mucosal or internal bleeding |
| Had any major surgery ≤4 weeks before screening |
| Had presence of active renal condition, except for those with isolated proteinuria due to MM |
| Had any serious and/or unstable pre-existing medical, psychiatric disorder or other conditions that could interfere with participation in the study |
| Had a current active liver or biliary disease (except for Gilbert's syndrome or asymptomatic gallstones, or otherwise stable chronic liver disease per investigator's assessment) |
| Had invasive malignancies, other than MM and curatively treated non-melanoma skin cancer, that required active therapy or were stable for <2 years |
| Had evidence of cardiovascular risk including ≥1 of the following:   - Current clinically significant untreated arrhythmias, including clinically significant electrocardiogram abnormalities such as 2nd degree (Mobitz Type II) or 3rd degree atrioventricular block - History of myocardial infarction, acute coronary syndromes (including unstable angina), coronary angioplasty, or stenting or bypass grafting ≤3 months before screening - Class III or IV heart failure as defined by the New York Heart Association functional classification system - Uncontrolled hypertension |
| Had a known immediate or delayed hypersensitivity reaction or idiosyncratic reaction to drugs chemically related to belantamab mafodotin, or any of the components of the study treatment |
| Had an active infection requiring treatment, or known HIV infection |
| Had presence of hepatitis B surface antigen, or hepatitis B core antibody  at screening or within 3 months before the first belantamab mafodotin dose |
| Had a positive hepatitis C antibody test result or positive hepatitis C RNA test result at  screening or ≤­3 months before the first belantamab mafodotin dose, except those with positive test results due to prior resolved disease |
| Had current corneal disease except mild punctate keratopathy |
| Were unable to tolerate antithrombotic prophylaxis |
| Discontinued prior treatment with lenalidomide due to intolerable adverse events |

*Without growth factor support for the past 14 days, excluding erythropoietin; ^†^isolated bilirubin 1.5 × ULN was acceptable if bilirubin was fractionated, and direct bilirubin was <35%; ^‡^calculated using the Modified Diet in Renal Disease formula.

eGFR, estimated glomerular filtration rate; FLC, free light chain; HIV, human immunodeficiency virus; LOT, line of therapy; MM, multiple myeloma; NCI-CTCAE, National Cancer Institute-Common Toxicity Criteria for Adverse Events; RNA, ribonucleic acid; SCT, stem cell transplant; ULN, upper limit of normal.

### **Table S2. Protocol-defined scale for grading ocular events associated with belantamab mafodotin**

| **Measure** | **Grade 1** | **Grade 2** | **Grade 3** | **Grade 4** |
| --- | --- | --- | --- | --- |
| Ophthalmic exam finding | Mild superficial keratopathy  (change from baseline) | Moderate punctate keratopathy and/or  mild/patchy microcysts  and/or mild/patchy epithelial or stromal  edema and/or  sub-epithelial haze (peripheral) and/or  active stromal opacity (peripheral) | Severe punctate keratopathy and/or  diffuse microcysts and/or  diffuse epithelial or stromal edema and/or sub-epithelial haze (central)  and/or active stromal opacity (central) | Corneal ulcer |
| Visual Acuity* | Change of 1 line from baseline | Change of 2–3 lines from baseline and not worse than 20/200 | Change of more than 3 lines from baseline and not worse than 20/200 | Worse than Vision 20/200 |

Grading was based on most severe finding; if eyes differed in severity, the protocol-defined grading should be based on the more severe eye. *Change in visual acuity due to ocular events, presented as Snellen equivalent and logarithm of the minimum angle of resolution value. If a change in vision was for a reason other than ocular events, the ophthalmic exam findings drove the event grading. If a patient had a baseline visual acuity of 20/200 or worse in an eye, ophthalmic exam findings drove the event grading.

### **Table S3. Patient demographics and clinical characteristics**

| **Parameter** | **1.9 mg/kg STRETCH (n=12)** | **1.9 mg/kg SINGLE (n=4)** | **2.5 mg/kg SPLIT  (n=13)** | **2.5 mg/kg SINGLE (n=16)** | **All-treated (N=45)** |
| --- | --- | --- | --- | --- | --- |
| **Age, years, median (range)** | 71.5  (48, 76) | 63.5  (53, 66) | 68.0  (36, 75) | 68.0  (55, 80) | 68.0  (36, 80) |
| ≥75, n (%) | 3 (25) | 0 | 1 (8) | 4 (25) | 8 (18) |
| **Male, n (%)** | 10 (83) | 4 (100) | 8 (62) | 13 (81) | 35 (78) |
| **Race, n (%)** |  |  |  |  |  |
| White |  |  |  |  |  |
| Caucasian/European heritage | 11 (92) | 4 (100) | 13 (100) | 13 (81) | 41 (91) |
| Arabic/North African heritage | 0 | 0 | 0 | 1 (6) | 1 (2) |
| Black/African American | 0 | 0 | 0 | 1 (6) | 1 (2) |
| Asian – Central/South Asian heritage | 0 | 0 | 0 | 1 (6) | 1 (2) |
| Native Hawaiian or Other Pacific Islander | 1 (8) | 0 | 0 | 0 | 1 (2) |
| **Prior LOT, median (range)** | 3.0 (1, 6) | 2.0 (1, 9) | 3.0 (1, 7) | 2.0 (1, 10) | 3.0 (1, 10) |
| **Prior MM treatment,* n (%)** |  |  |  |  |  |
| Corticosteroids | 12 (100) | 4 (100) | 13 (100) | 15 (94) | 44 (98) |
| Chemotherapy | 11 (92) | 4 (100) | 13 (100) | 14 (88) | 42 (93) |
| Proteasome inhibitor | 10 (83) | 4 (100) | 13 (100) | 15 (94) | 42 (93) |
| Immunomodulator | 11 (92) | 3 (75) | 9 (69) | 14 (88) | 37 (82) |
| Lenalidomide | 7 (58) | 1 (25) | 8 (62) | 10 (63) | 26 (58) |
| Monoclonal antibody | 3 (25) | 1 (25) | 7 (54) | 5 (31) | 16 (36) |
| Daratumumab | 3 (25) | 1 (25) | 5 (38) | 5 (31) | 14 (31) |
| Other^‡^ | 0 | 0 | 1 (8) | 3 (19) | 4 (9) |
| **Prior stem cell transplant, n (%)** | 7 (58) | 4 (100) | 9 (69) | 13 (81) | 33 (73) |
| **ISS Stage, n (%)** |  |  |  |  |  |
| I | 6 (50) | 4 (100) | 5 (38) | 9 (56) | 24 (53) |
| II | 5 (42) | 0 | 4 (31) | 3 (19) | 12 (27) |
| III | 1 (8) | 0 | 4 (31) | 2 (13) | 7 (16) |
| Unknown | 0 | 0 | 0 | 2 (13) | 2 (4) |
| **ECOG performance score, n (%)** |  |  |  |  |  |
| 0 | 5 (42) | 4 (100) | 4 (31) | 5 (31) | 18 (40) |
| 1 | 7 (58) | 0 | 9 (69) | 9 (56) | 25 (56) |
| 2 | 0 | 0 | 0 | 2 (13)^†^ | 2 (4) |
| **Extramedullary disease present, n (%)** | 2 (17) | 0 | 1 (8) | 3 (19) | 6 (13) |
| **Myeloma Ig, n (%)** |  |  |  |  |  |
| IgA | 3 (25) | 0 | 2 (15) | 2 (13) | 7 (16) |
| IgG | 11 (92) | 3 (75) | 11 (85) | 12 (75) | 37 (82) |
| IgM | 1 (8) | 0 | 0 | 0 | 1 (2) |
| Not applicable | 0 | 1 (25) | 0 | 2 (13) | 3 (7) |
| **Myeloma light chain, n (%)** |  |  |  |  |  |
| No | 3 (25) | 0 | 5 (38) | 3 (19) | 11 (24) |
| Yes: Kappa light chain | 7 (58) | 3 (75) | 7 (54) | 10 (63) | 27 (60) |
| Yes: Lambda light chain | 2 (17) | 1 (25) | 1 (8) | 3 (19) | 7 (16) |
| **Cytogenetics,**^†,§^ **n (%)** |  |  |  |  |  |
| High risk | 2 (17) | 0 | 6 (46) | 6 (38) | 14 (31) |
| t(4;14)^§^ | 2 (100) | 0 | 3 (50) | 1 (17) | 6 (43) |
| t(14;16)^§^ | 0 | 0 | 0 | 1 (17) | 1 (7) |
| 17p13del^§^ | 1 (50) | 0 | 4 (67) | 4 (67) | 9 (64) |
| Other^¶^ | 10 (83) | 4 (100) | 7 (54) | 10 (63) | 31 (69) |
| 1q21+^#^ | 2 (20) | 0 | 5 (71) | 4 (40) | 11 (35) |
| Del 1p^#^ | 0 | 0 | 3 (43) | 1 (10) | 4 (13) |
| **Hypogammaglobulinemia,^\|\|^ n (%)** | 0 | 0 | 1 (8) | 0 | 1 (2) |

Parameters were assessed at screening. *Patients may be included in more than one category; ^†^earlier versions of the study protocol allowed for patients to be enrolled with an ECOG score >1; ^‡^includes uncoded medication; ^§^percentages based on the number of patients included in the high-risk cytogenetics category; ^¶^includes non-high risk, negative, not evaluated, or not done options; ^#^percentages based on the number of patients included in the Other cytogenetics category; ^||^physician designated.

ECOG; Eastern Cooperative Oncology Group; Ig, immunoglobulin; ISS, International Staging System; LOT, line of therapy; MM, multiple myeloma.

### **Table S4. Minimal residual disease negativity by response**

| **Best confirmed response** | **1.9 mg/kg STRETCH (n=12)** | **1.9 mg/kg SINGLE (n=4)** | **2.5 mg/kg SPLIT  (n=13)** | **2.5 mg/kg SINGLE (n=16)** |
| --- | --- | --- | --- | --- |
| **sCR/CR/VGPR** |  |  |  |  |
| No. of patients with MRD data | 3 | 1 | 3 | 7 |
| MRD negativity rate, n (%) [95% CI]* | 2 (17)  [2.1, 48.4] | 1 (25)  [0.6, 80.6] | 3 (23)  [5.0, 53.8] | 4 (25)  [7.3, 52.4] |
| **sCR/CR** |  |  |  |  |
| No. of patients with MRD data | 3 | 1 | 2 | 6 |
| MRD negativity rate, n (%) [95% CI]* | 1 (8) [0.2, 38.5] | 1 (25) [0.6, 80.6] | 1 (8) [0.2, 36.0] | 4 (25) [7.3, 52.4] |

*Percentages based on the all-treated population.
CI, confidence interval; CR, complete response; MRD, minimal residual disease; No., number; sCR, stringent complete response; VGPR, very good partial response

### **Figure S1. DREAMM-6 Arm A study design
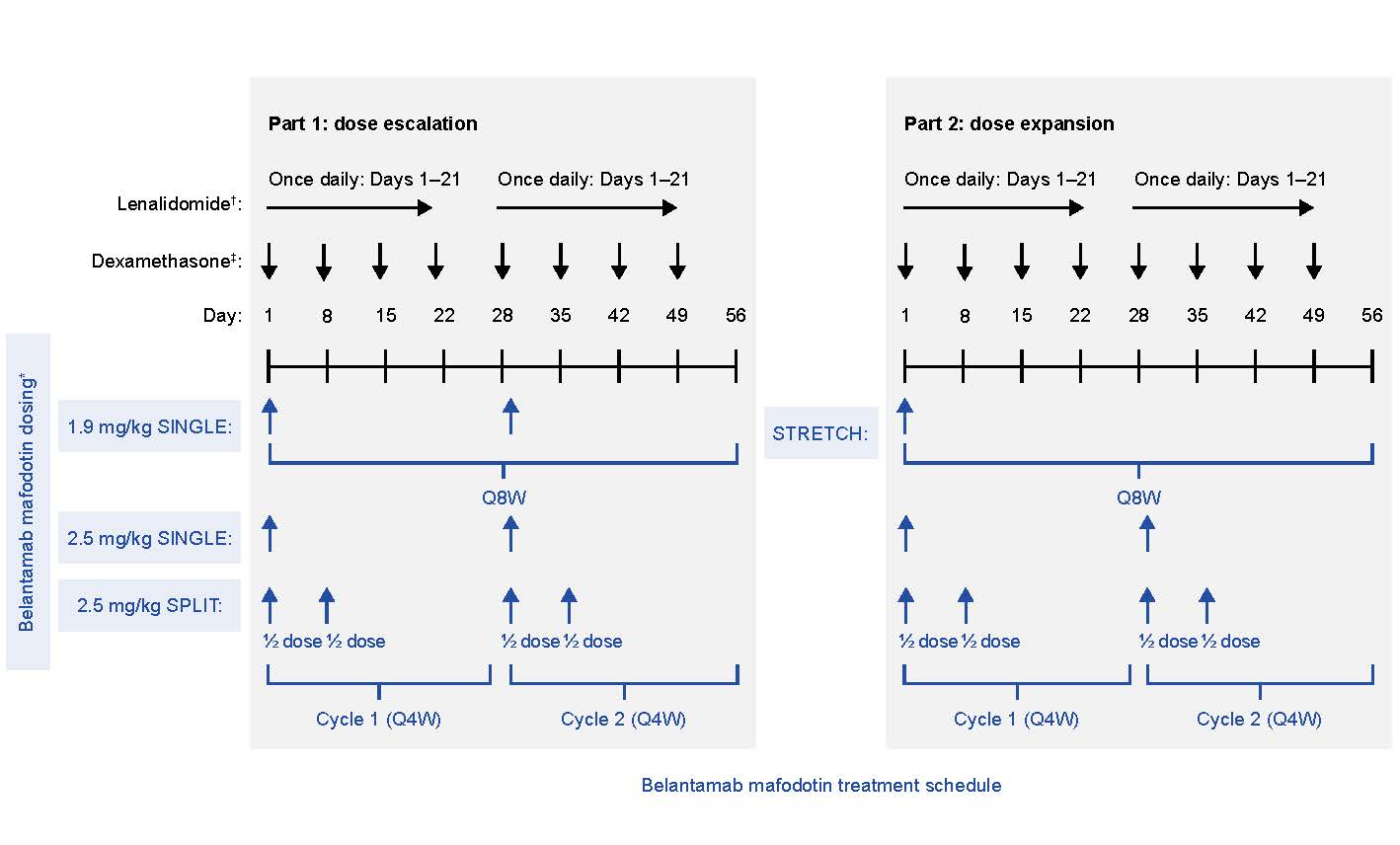
**

*The 2.5 mg/kg (IV) dose was the starting dose, with the 1.9 mg/kg as an optional lower dose if the 2.5 mg/kg dose was not tolerated; ^†^patients with eGFR ≥60 mL/min/1.73m^2^ received 25 mg/day (PO) and those with eGFR 40–<60 mL/min/1.73m^2^ received 10 mg/day (PO); ^‡^administered PO (40 mg). Patients aged >75 years with a body mass index <18.5 kg/m^2^ were eligible to receive a reduced dose (20 mg).

eGFR; estimated glomerular filtration rate; IV, intravenous; PO, orally; Q4W, every 28 days; Q8W, every 8 weeks.

### **Figure S2. Patient disposition**

**
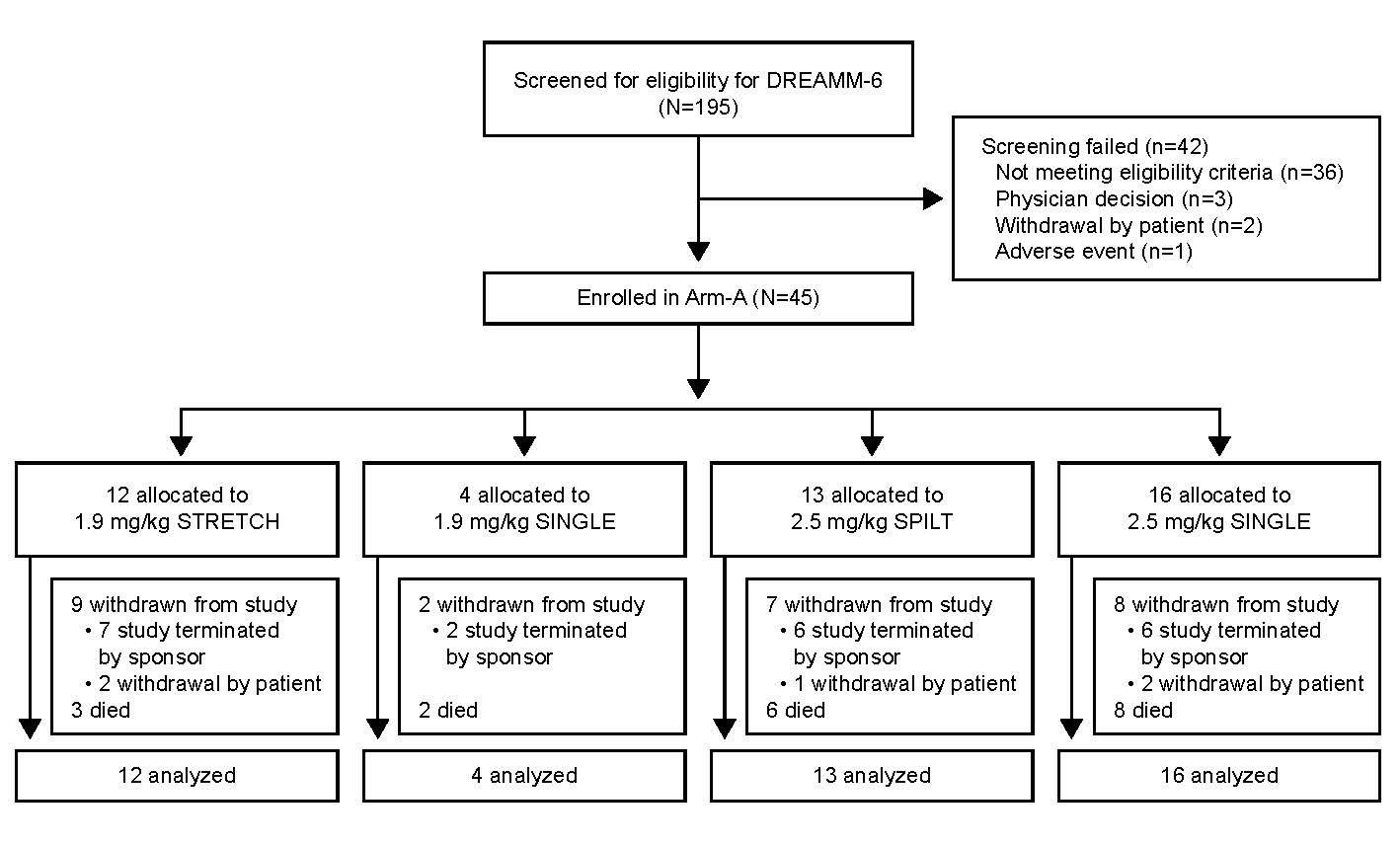
**
